# Active Tuberculosis among Homeless Persons, Toronto, Ontario, Canada, 1998–2007

**DOI:** 10.3201/eid1703.100833

**Published:** 2011-03

**Authors:** Kamran Khan, Elizabeth Rea, Cameron McDermaid, Rebecca Stuart, Catharine Chambers, Jun Wang, Angie Chan, Michael Gardam, Frances Jamieson, Jae Yang, Stephen W. Hwang

**Affiliations:** Author affiliations: St. Michael’s Hospital, Toronto, Ontario, Canada (K. Khan, C. Chambers, J. Wang, A. Chan, J. Yang, S.W. Hwang);; University of Toronto, Toronto (K. Khan, E. Rea, M. Gardam, F. Jamieson, S.W. Hwang);; Toronto Public Health, Toronto (E. Rea, R. Stuart);; City of Ottawa, Ottawa, Ontario, Canada (C. McDermaid);; University Health Network, Toronto (M. Gardam);; Ontario Agency for Health Promotion and Protection, Toronto (F. Jamieson)

**Keywords:** Tuberculosis, homeless persons, epidemiology, molecular epidemiology, clinical medicine, tuberculosis and other mycobacteria, research

## Abstract

While tuberculosis (TB) in Canadian cities is increasingly affecting foreign-born persons, homeless persons remain at high risk. To assess trends in TB, we studied all homeless persons in Toronto who had a diagnosis of active TB during 1998–2007. We compared Canada-born and foreign-born homeless persons and assessed changes over time. We identified 91 homeless persons with active TB; they typically had highly contagious, advanced disease, and 19% died within 12 months of diagnosis. The proportion of homeless persons who were foreign-born increased from 24% in 1998–2002 to 39% in 2003–2007. Among foreign-born homeless persons with TB, 56% of infections were caused by strains not known to circulate among homeless persons in Toronto. Only 2% of infections were resistant to first-line TB medications. The rise in foreign-born homeless persons with TB strains likely acquired overseas suggests that the risk for drug-resistant strains entering the homeless shelter system may be escalating.

In Canada’s major cities, tuberculosis (TB) is increasingly becoming a disease of persons born outside Canada (foreign-born). In 2009 in the city of Toronto in Ontario, 94% of all persons with active TB were foreign-born (*1*). Although homeless and marginally housed persons represent a smaller proportion of TB case-patients, they remain a persistent high-risk population. Recent TB outbreaks and disease clusters among homeless persons have been reported in many cities in the United States (*2*–*5*) and have been associated with transmission at shelters, single-room–occupancy hotels, and rooming houses (which provide inexpensive rooms with shared bathrooms), prisons, and bars (*6*–*10*).

Toronto is the largest city in Canada; among its population of 2.5 million persons, ≈50% were born outside Canada (*11*). Each year in Toronto, ≈29,000 persons use emergency shelters, and on any given night ≈5,000 are without homes (*12*). During 2001–2002, a large shelter-based TB outbreak occurred among homeless persons in Toronto. A coroner’s inquest into the death of a homeless man in whom pulmonary TB developed during the course of this outbreak revealed the many challenges of diagnosing and managing TB in homeless populations (*13*). In response to the inquest and resulting jury recommendations, major changes to the management of homeless TB cases occurred in the public health and shelter systems, and local TB clinic capacity expanded. This case resulted in the creation of a public health team dedicated to case management, contact follow-up, advocacy, education, health promotion, and active case finding among the city’s homeless and underhoused population.

A comprehensive review of the population and molecular epidemiology, clinical features, management and health outcomes of homeless persons with TB in Canada is needed but lacking. To better understand and address the extent of disease in this vulnerable population, we studied TB among Toronto’s homeless persons over a 10-year period.

## Methods

The study population included all persons in Toronto for whom active TB had been reported to Toronto Public Health from January 1, 1998, through December 31, 2007. Data were extracted from the Reportable Disease Information System and the Integrated Public Health Information System for all case-patients with a risk setting of “shelter/rooming house” or a risk factor of “homeless.” Health case management files were reviewed to ensure accuracy of database entries; additional data were abstracted when necessary. Cases were included in the analysis for persons with active TB who met the following eligibility criteria in the year before diagnosis: 1) any shelter stay, 2) any rooming house stay, 3) no fixed address, or 4) use of services for homeless persons >1× per week. Cases were excluded for persons with active TB who 1) were foreign-born and received a diagnosis of active TB within 1 month of arrival in Canada, 2) received a diagnosis of active TB while in a shelter designed exclusively for resettlement of newly arrived refugees, 3) were not residents of Toronto when they received a diagnosis of active TB, or 4) had incomplete records.

We collected data on patient demographics, clinical features of TB disease, medical management and health outcomes of patients, and molecular fingerprinting of the TB bacterium. Case types were classified according to the Public Health Agency of Canada definition of new and re-treatment TB cases (*14*). All chest radiographs were interpreted by radiologists. Susceptibility testing for *Mycobacterium tuberculosis* was performed at the Central Public Health Laboratory of the Ontario Agency for Health Protection and Promotion. All isolates from new TB cases were tested for susceptibility to first-line drugs (isoniazid, rifampin, pyrazinamide, and ethambutol) according to recommended standard protocols, by using the commercial broth system, BACTEC MGIT 960 (Becton, Dickinson and Company, Sparks, MD, USA). Isolates resistant to rifampin or any 2 first-line drugs were also tested for susceptibility to second-line drugs (*15*). Restriction fragment-length polymorphism was performed for strain genotyping by using established methods (*16*). Genotypes were analyzed by using Bionumerics 5.0 (Applied Maths, Saint-Martens Latem, Belgium). HIV test results were recorded when available; information about use of antiretroviral therapy for HIV/TB–co-infected patients was not available.

Comparisons were made between Canada-born and foreign-born case-patients and between 5-year periods (1998–2002 and 2003–2007) using the 2-sided Fisher exact test or χ^2^ test, as appropriate. Kaplan-Meier plots were generated to determine time to death from all causes during the 12 months after TB diagnosis. The log-rank test was used to compare survival curves between the 2 groups. Because of the small cohort size, multivariate regression analyses were not performed. All analyses were performed by using SAS version 9.1.3 (SAS Institute, Cary, NC, USA). Ethics approval was obtained from St. Michael’s Hospital Research Ethics Board.

## Results

**Figure 1:**
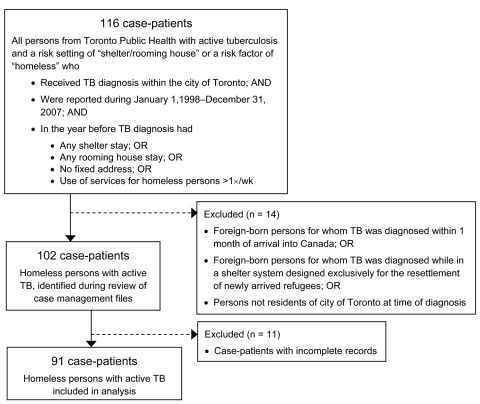
Inclusion–exclusion criteria for study of active tuberculosis (TB) in homeless persons, Toronto, Ontario, Canada, 1998–2007.

**Figure 2:**
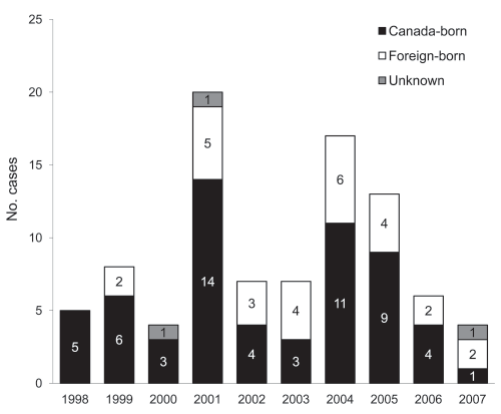
Number of reported cases of active tuberculosis in homeless persons, Toronto, Ontario, Canada, 1998–2007.

From January 1, 1998, through December 31, 2007, a total of 3,685 active TB cases were reported to Toronto Public Health; among these, 102 (2.8%) met the study inclusion criteria. Incomplete records for 11 patients resulted in a final sample size of 91 (Figure 1). Most patients were absolutely homeless (i.e., living on the street or in a shelter); 86 (95%) patients reported staying in a shelter, having no fixed address, and/or using services for homeless persons >1× per week. Five (5%) patients did not fall into any of these categories but had lived in a rooming house during the past year. Birthplace was available for 88 patients; nearly one third (n = 28; 32%) were born outside Canada (Table 1). The proportion of foreign-born patients increased over time from 24% (n = 10) in 1998–2002 to 39% (n = 18) in 2003–2007 (Table 2). Among the Canada-born homeless persons with TB, 13 (22%) were Aboriginal. The number of reported cases of active TB over the study period by place of birth is shown in Figure 2. Approximately equal numbers of cases were reported during the 2 periods: 44 (48%) during 1998–2002 and 47 (52%) during 2003–2007.

**Table 1:** Country of origin for 28 foreign-born homeless persons with tuberculosis, Toronto, Ontario, Canada, 1998–2007

Country	No.
Burundi	1
Chile	1
China*	1
Costa Rica	1
Ethiopia*	2
Germany	1
Guyana	2
India*	2
Iraq	1
Ireland	2
Nepal	1
Nigeria*	1
Philippines*	1
Poland	1
Somalia	2
Tanzania*	1
Tibet	1
Turkey	1
Uganda*	2
United Kingdom	1
Yemen	1
Former Yugoslavia	1
Zimbabwe*	2

*1 of 22 countries that account for 80% of all new of tuberculosis cases annually (high-burden countries).

**Table 2:** Demographic and clinical characteristics of 91 homeless persons with tuberculosis, Toronto, Ontario, Canada, 1998–2007*

Characteristic	All persons, no. (%)	Canada born, no. (%)	Foreign born, no. (%)	p value†	1998–2002, no. (%)	2003–2007, no. (%)	p value‡
Median age, y (IQR)	47 (38–56)	49 (42–58)	38 (30–50)	–	45 (38–59)	48 (40–54)	–
Male sex	81 (89)	53 (88)	25 (89)	1.00	40 (91)	41 (87)	0.74
Origin							0.15
Canada born, not Aboriginal	47 (53)	47 (78)	NA		27 (64)	20 (43)	
Canada born, Aboriginal	13 (15)	13 (22)	NA		5 (12)	8 (17)	
Foreign born	28 (32)	NA	28 (100)		10 (24)	18 (39)	
Case type				0.43			1.00
New	83 (91)	53 (88)	27 (96)		40 (91)	43 (91)	
Re-treated§	8 (9)	7 (12)	1 (4)		4 (9)	4 (9)	
Method of detection				–			–
Signs and symptoms	51 (56)	30 (50)	18 (64)		23 (52)	28 (60)	
Contact tracing	19 (21)	16 (27)	3 (11)		11 (25)	8 (17)	
Diagnosis while under care for other condition	8 (9)	8 (13)	0		4 (9)	4 (9)	
Immigration screening	6 (7)	NA	6 (21)		6 (14)	0	
Active case finding (sputum screening)	3 (3)	3 (5)	0		NA	3 (6)	
Jail	1 (1)	1 (2)	0		0	1 (2)	
Other¶	3 (3)	2 (3)	1 (4)		0	3 (6)	
Site(s) of infection				1.00			0.15
Pulmonary only	67 (74)	45 (75)	21 (75)		33 (75)	34 (72)	
Extrapulmonary only	21 (23)	13 (22)	6 (21)		8 (18)	13 (28)	
Pulmonary and extrapulmonary	3 (3)	2 (3)	1 (4)		3 (7)	0	
Chest radiograph at diagnosis#							0.08
No abnormalities	9 (13)	7 (15)	2 (9)	0.86	7 (19)	2 (5)	
Abnormal without cavitation	45 (65)	30 (64)	15 (68)		24 (67)	21 (62)	
Abnormal with cavitation	15 (22)	10 (21)	5 (23)		5 (14)	11 (32)	
Self-reported symptoms	73 (80)	50 (83)	20 (71)	0.26	34 (77)	39 (83)	0.60
Median time from symptom onset to diagnosis, mo (IQR)	1.9 (0.6–3.1)	1.8 (0.6–2.6)	2.6 (0.8–5.8)	–	1.8 (0.6–3.1)	2.2 (0.6–3.2)	–
Sputum smear results at diagnosis**				0.39			0.58
Negative	21 (33)	12 (29)	9 (43)		12 (40)	9 (27)	
Scarce/moderate	13 (21)	8 (20)	5 (24)		5 (17)	8 (24)	
Numerous	29 (46)	21 (51)	7 (33)		13 (43)	16 (49)	
Method of diagnosis				0.59			0.18
Positive culture	86 (95)	55 (92)	28 (100)		40 (91)	46 (98)	
Positive AMTD	3 (3)	3 (5)	0		3 (7)	0	
Clinical	2 (2)	2 (3)	0		1 (2)	1 (2)	

*Birthplace information available for 88 persons. IQR, interquartile range; NA, not applicable; –, no statistical test performed; AMTD, amplified *Mycobacterium tuberculosis* direct test. †Probability of a significant difference between Canada-born and foreign-born persons for each variable; calculated by using the 2-sided Fisher exact test or χ^2^ test, as appropriate. ‡Probability of a significant difference between the 2 periods for each variable; calculated by using the 2-sided Fisher exact test or χ^2^ test, as appropriate. §Public Health Agency of Canada definition: documented evidence or adequate history of previously active tuberculosis (TB) that was declared cured or treatment completed by current standards, AND at least 6 mo have passed since the last day of previous treatment, AND a diagnosis with a subsequent episode of TB that meets the active TB case definition. ¶Includes shelter screening, routine screening at other centers, and other detection methods. #Results for only the 70 persons with pulmonary disease. **Sputum smears available for 63 patients with pulmonary disease.

Demographic information, clinical characteristics, and concurrent medical conditions for patients are presented in Tables 2 and 3. Homeless persons with TB were often highly contagious at the time of diagnosis, as demonstrated by the large proportion of patients who had cavitating pulmonary disease and sputum smears with numerous acid-fast bacilli. The median duration of symptoms for persons with pulmonary disease before diagnosis was 2.5 months (interquartile range 0.6–3.1 months). Pulmonary disease was found in 67 (74%) patients, among whom 29 (46%) showed numerous acid-fast bacilli in sputum smear. The proportion of pulmonary TB patients with cavitary disease increased over time from 14% (n = 5) in 1998–2002 to 32% (n = 11) in 2003–2007.

**Table 3:** Concurrent conditions of 91 homeless persons with tuberculosis, Toronto, Ontario, Canada, 1998–2007*

Condition	All persons, no. (%)	Canada born, no. (%)	Foreign born, no. (%)	p value†	1998–2002, no. (%)	2003–2007, no. (%)	p value‡
HIV infection				0.52			0.33
Positive	11 (12)	9 (15)	1 (4)		5 (11)	6 (13)	
Negative	6 (7)	4 (7)	2 (7)		1 (2)	5 (10)	
Unknown	74 (81)	47 (78)	25 (89)		38 (87)	36 (77)	
Psychiatric disease§	10 (11)	7 (12)	2 (7)	0.71	2 (5)	8 (17)	0.09
COPD	8 (9)	7 (12)	0	0.09	6 (14)	2 (4)	0.15
Liver disease¶	29 (32)	24 (40)	4 (14)	0.03	12 (27)	17 (36)	0.38
Cancer	5 (5)	4 (7)	1 (4)	1.00	3 (7)	2 (4)	0.67
Congestive heart failure	1 (1)	0	0	–	1 (2)	0	0.48
Diabetes	11 (12)	5 (8)	5 (18)	0.28	1 (2)	10 (21)	0.01
Chronic alcohol abuse	29 (32)	23 (38)	6 (21)	0.15	12 (27)	17 (36)	0.38
Injection drug use	12 (13)	10 (17)	1 (4)	0.16	6 (14)	6 (13)	1.00
Noninjection drug use	6 (7)	6 (10)	0	0.17	0	6 (13)	0.03
Other	1 (1)	0	1(4)	0.32	0	1 (2)	1.00

*Birthplace information available for 88 persons.–, no statistical test performed; COPD, chronic obstructive pulmonary disease. †Probability of a significant difference between Canada-born and foreign-born persons for each variable; calculated by using the 2-sided Fisher exact test or χ^2^ test, as appropriate. ‡Probability of a significant difference between the 2 periods for each variable; calculated by using the 2-sided Fisher exact test or χ^2^ test, as appropriate. §Includes schizophrenia, severe mental illness, and dementia. ¶Includes cirrhosis, viral hepatitis B or C.

In terms of treatment information and outcomes, 75% of homeless persons with TB started treatment within 4 days of diagnosis (median 1 day; interquartile range 0–4 days) (Table 4). Most patients received closely monitored treatment within hospitals or as outpatients under directly observed therapy (DOT) (median treatment duration 2.0 and 6.2 months, respectively); few received self-administered therapy for any substantial period of time. Only 1 patient required a court order for treatment in a TB sanitarium.

**Table 4:** Treatment-associated characteristics of 91 homeless persons with tuberculosis, Toronto, Ontario, Canada, 1998–2007*

Characteristic	All persons, no. (%)	Canada-born, no. (%)	Foreign born, no. (%)	p value†	1998–2002, no. (%)	2003–2007, no. (%)	p value‡
Days from diagnosis to initiation of treatment, median (IQR)	1 (0–4)	0.5 (0–4)	1 (0–6)	–	1 (0–3)	1 (0–4)	–
Duration of treatment, mo, median (IQR)							
Total	9.9 (6.5–12.7)	10.0 (6.8–12.5)	8.6 (6.0–12.9)	–	9.0 (6.1–12.2)	10.8 (6.7–13.1)	–
Treatment in institution	2.0 (0.2–4.0)	2.4 (0.5–3.9)	0.6 (0–4.2)		0.9 (0.2–3.9)	2.2 (0.0–4.1)	
Treatment under directly observed therapy	6.2 (0.8–8.8)	6.4 (0.9–9.0)	6.0 (2.4–7.2)		5.7 (0.2–7.0)	6.6 (4.5–9.0)	
Treatment by self- administered therapy	0.1 (0.1–0.9)	0.1 (0.1–0.6)	0.3 (0.1–2.1)		0.2 (0.0–1.1)	0.1 (0.1–0.6)	
Admission to hospital	76 (84)	55 (92)	19 (68)	0.01	35 (80)	41 (87)	0.40
Treatment under public health order§	15 (16)	9 (15)	6 (21)	0.55	4 (9)	11 (23)	0.09
Court-ordered detention for treatment¶	1 (1)	1 (2)	0	–	1 (2)	0	–
Outcome#				0.13			0.74
Treatment completed	70 (78)	45 (75)	25 (93)		33 (75)	37 (80)	
Died while receiving treatment**	17 (19)	12 (20)	2 (7)		9 (20)	7 (16)	
Lost to follow-up	2 (2)	2 (3)	0		1 (2)	1 (2)	
Refused further care	1 (1)	1 (2)	0		1 (2)	0	

*Birthplace information available for 88 persons. IQR, interquartile range; –, no statistical test performed. †Probability of a significant difference between Canada-born and foreign-born persons for each variable; calculated by using the 2-sided Fisher exact test or χ^2^ test, as appropriate. ‡Probability of a significant difference between the 2 periods for each variable; calculated by using the 2-sided Fisher exact test or χ^2^ test, as appropriate. §Referred to in Ontario as a Section 22. ¶Referred to in Ontario as a Section 35. #1 patient continues treatment at the time of this report. **One foreign-born case-patient from 2003–2007 period died 14 mo after onset of treatment. Study considers death from all causes within 12 mo of TB diagnosis. After 12 mo, causes of death other than TB become more relevant; therefore, deaths occurring after 12 mo are not included in this estimate.

**Figure 3:**
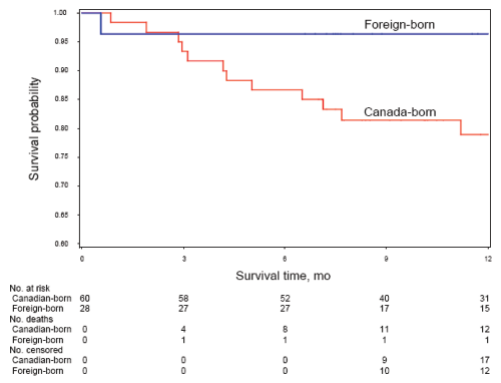
Probability of death from all causes during the 12-month period after tuberculosis diagnosis among Canada-born and foreign-born homeless persons with active tuberculosis, Toronto, Ontario, Canada, 1998–2007. Birthplace information available for 88 persons. Censored are patients who completed treatment for tuberculosis or were lost to follow up.

Almost 1 of 5 (n = 17; 19%) patients died (from any cause) within 12 months of diagnosis (Table 4); most (n = 12; 86%) patients who died were born in Canada; 4 were HIV positive, 1 was HIV negative, and the remaining 12 had unknown HIV status. Among patients who survived, most (n = 70; 96%) homeless persons with TB successfully completed treatment and only 3 were lost to follow-up or refused further care. Probability of survival during the 12 months after diagnosis was lower for Canada-born versus foreign-born homeless persons (p = 0.06; Figure 3). No changes in survival probabilities were seen between the 2 periods, 1998–2002 and 2003–2007 (data not shown).

TB isolates were genotyped and tested for antimicrobial drug resistance (Table 5). Of the 4 strains known to circulate among Toronto’s homeless population (A, B, C, or D), isolates from ≈90% of Canada-born patients belonged to one of these strains, and isolates from >50% of foreign-born patients belonged to none of them. The proportion of isolates not belonging to these 4 strains increased over time, from 14% (n = 4) during 1998–2002 to 32% (n = 12) during 2003–2007. Almost all (n = 84; 98%) isolates were susceptible to first-line TB medications. Only 2 isolates demonstrated evidence of antimicrobial drug resistance: 1, from a Canada-born patient, was resistant to ethambutol only, and 1, from a foreign-born patient, was resistant to isoniazid only.

**Table 5:** Characteristics of isolates from 91 homeless persons with tuberculosis, Toronto, Ontario, Canada, 1998–2007*

Characteristic	Culture-confirmed case, no. (%)	Canada-born, no. (%)	Foreign born, no. (%)	p value†	1998–2002, no. (%)	2003–2007, no. (%)	p value‡
RFLP type				<0.001			0.13
Strain A	32 (48)	25 (52)	6 (38)		17 (59)	15 (41)	
Strain B	8 (12)	7 (15)	1 (6)		3 (10)	5 (14)	
Strain C	7 (11)	7 (15)	0		5 (17)	2 (5)	
Strain D	3 (5)	3 (6)	0		0	3 (8)	
Other strain	16 (24)	6 (13)	9 (56)		4 (14)	12 (32)	
Not tested	20	7	12		11	9	
Antimicrobial drug resistance				1.00			0.21
No	84 (98)	54 (98)	27 (96)		38 (95)	46 (100)	
Yes§	2 (2)	1 (2)	1 (4)		2 (5)	0	

*Birthplace information available for 88 persons. RFLP, restriction fragment-length polymorphism. †Probability of a significant difference between Canada-born and foreign-born persons for each variable; calculated by using the 2-sided Fisher exact test or χ^2^ test, as appropriate. ‡Probability of a significant difference between the 2 periods for each variable; calculated by using the 2-sided Fisher exact test or χ^2^ test, as appropriate. §Represents 1 Canada-born person whose isolate was resistant to ethambutol only and 1 foreign-born case whose isolate was resistant to isoniazid only.

## Discussion

Homeless persons in our cohort received nearly all health care services for TB in hospital or under careful observation in the outpatient environment. All outpatients received DOT, were accompanied by public health staff to all clinic visits, and received intensive social assistance. Despite these efforts, all-cause mortality rates for our cohort were extremely high. Among Canada-born homeless persons with TB in our study, 20% died within 1 year of their diagnosis; in comparison, among all persons with TB in Toronto during 1999–2002, only 7.4% died (*17*). All-cause mortality rates for homeless populations in general are disproportionately high; rates among men who use shelters in Toronto are 2–8× higher than rates for the general population (*18*). Homeless persons often have more concurrent medical conditions (e.g., HIV, liver disease), mental health conditions (e.g., schizophrenia), and/or dependence on substances, any of which may raise their risk for primary or reactivated TB, complicate delivery of health services, and negatively affect treatment outcomes.

Our findings reflect the increased rates of illness and death among homeless persons and suggest that urgent measures are needed to improve TB treatment outcomes for this vulnerable population. Recent research suggests that homeless immigrants in Toronto are in general healthier and possess fewer concurrent illnesses than Canada-born homeless persons, which may explain the lower prevalence of concurrent illnesses among foreign-born TB patients and the differences in mortality rates according to place of birth (*19*).

Homeless TB patients represent ≈3% of all TB patients in Toronto, of which a growing proportion are foreign-born, likely reflecting the changing demographics in the city overall and in the homeless population itself. Our findings suggest that Canada-born patients with TB were more likely to be infected with strains known to circulate within shelters and other social networks in Toronto. In contrast, active TB in foreign-born patients was more likely to result from reactivation of latent infection with strains acquired overseas (*20*,*21*).

To date, drug resistance among homeless TB patients is rare; laboratory evidence of drug resistance was demonstrated for only 2% of homeless TB patients compared with 14% of all culture-positive TB patients in Toronto (*1*). Because being born outside Canada is a known risk factor for drug-resistant TB, the rise in foreign-born homeless TB patients and the corresponding increase in heterogeneity of strain genotypes are concerning and may pose serious and growing threats to the homeless shelter system (*20*,*22*–*25*). The outbreaks of multidrug resistant TB in New York City during the 1980s and early 1990s highlight the potential dangers of introducing drug-resistant infections into the shelter system and call for increased prevention and control efforts (*26*).

Despite the increase in foreign-born homeless persons with TB over time, most homeless TB patients in our sample were Canada-born (68%), a substantial proportion of whom were of Aboriginal origin (22%). By comparison, in 2008, only 6% of persons with active TB in Toronto were Canada-born (*1*). Although TB in Canada is primarily a disease of foreign-born persons (*14*), our results suggest that TB transmission persists among Canada-born inner city homeless populations. These findings also underscore the need to address TB transmission within the homeless shelter system. Furthermore, the disproportionately high prevalence of Canada-born Aboriginal persons in our sample suggests that further efforts are needed to address the high incidence of TB in this population.

Homeless TB patients tend to seek care when disease is advanced and highly contagious, defined by abnormal chest radiographic findings (cavitation) and numerous acid-fast bacilli in sputum smear. Although many homeless patients had pulmonary disease, the number was proportional to the prevalence of pulmonary disease among all TB patients in Toronto (*1*). In patients with pulmonary TB, nearly half had numerous acid-fast bacilli in sputum smear. An increasing prevalence of cavitary disease on chest radiographs was observed over time, despite increasing intervention and active case-finding initiatives during the more recent 5-year period of this study. However, the increase in cavitary disease could be related to delays in seeking health care, as indicated by the increase in median time from symptom onset to diagnosis over the 2 periods of the study.

Homeless TB patients often have difficulty accessing the health care system and may prioritize subsistence needs such as food and shelter over health services, especially those perceived as discretionary (*27*). These factors, as well as cultural and language barriers among foreign-born patients (*28*,*29*), may contribute to delays in seeking health care, which lead to advanced disease and hospitalization (*27*,*30*,*31*). For our sample, hospitalization rates were high; >80% of patients were hospitalized. This is noteworthy in Canada, where most TB patients are treated as outpatients, even at the time of diagnosis (*32*). The inability to isolate infectious homeless patients in outpatient settings such as shelters largely explains the high rate of hospitalization for patients in our sample.

Adherence to treatment is often challenging for patients who are homeless or living in transient, substandard housing and who may have concurrent substance use or mental health problems. Consequently, DOT is usually implemented for homeless persons with TB in Canada (*32*). In the province of Ontario, all patients with active TB are eligible to receive either inpatient or outpatient TB treatment, regardless of their insurance coverage. Most patients in our sample received their entire treatment closely monitored within hospitals, with outpatient DOT, or both. A few patients self-administered treatment for short periods. Despite the common perception that homeless TB patients are noncompliant with treatment (*33*–*35*), ≈80% of patients in our sample completed treatment, which is equivalent to the treatment completion rate for all TB patients in Toronto receiving DOT (*1*). Intensive case management by public health and clinic staff as well as small incentives and enablers (e.g., food vouchers or cash) helped ensure high completion rates for this population. For most homeless TB patients who did not complete treatment, the reason was that they died; only a few were lost to follow-up or refused further care.

The strength of this study is that it provides a comprehensive description of all cases of TB among homeless persons in a large, ethnically diverse city in Canada over a 10-year period. However, the study also has limitations. Only patients who were residents of the city of Toronto at the time of diagnosis were included in the analysis; consequently, homeless persons with active TB who may have been exposed in Toronto shelters or rooming houses and later moved elsewhere were not detected. Furthermore, homeless persons with TB were not included in the analysis if they had a history of shelter use >1 year before diagnosis with active TB. As a result, some patients who acquired TB infection while homeless but who subsequently acquired housing may have been missed. Furthermore, our retrospective study used public health surveillance data; consequently, our analyses are subject to limitations in how the data were originally collected. We excluded 14 patients with active TB because their records were incomplete; hence, we were unable to determine whether they differed demographically from included patients. This limitation could have influenced the results of our analyses that were stratified by birthplace.

Molecular fingerprinting data were unavailable for 20 isolates, which may have influenced the genotyping trends we observed over time. Because we were unable to definitively determine the number of patients who died directly or indirectly as a result of TB, the mortality rates represent death from all causes in the year after TB diagnosis. Although our study was conducted in a single metropolitan urban center, our findings and recommendations may be relevant to other large cities where levels of immigration and poverty are high.

Prior research among homeless persons in New York City shows decreasing trends in rates of active TB during 1992–2006 and demonstrates that public health prevention and control efforts (e.g., latent TB infection screening) in this population can be effective (*36*). In our study, several homeless patients were originally identified as tuberculin skin test–positive contacts before active TB developed, but they were unwilling to start treatment for latent TB infection, were deemed poor candidates for treatment because of serious underlying medical or mental health conditions, or could not complete a course of treatment because of adverse drug reactions. Lack of treatment for latent TB occurred despite substantial incentives and enablers for persons to initiate and continue therapy (e.g., cash for attending clinic visits, free passes for taxis or public transit, use of DOT for latent TB infection). Hence, for this cohort the opportunity to mitigate the risk for development of active TB through the treatment of latent TB infection was limited by the above challenges.

Primary prevention efforts should focus on shelter-based control measures, which have proven effective at reducing person-to-person spread of drug-resistant TB in other urban centers (*37*). Improved ventilation systems at shelters will help reduce the spread of TB during an outbreak (*38*,*39*). Smaller shelter sizes and strategies to reduce mobility (e.g., eliminating length of stay restrictions at shelters) may also help limit the extent of transmission. Additionally, expansion of sustainable housing programs for homeless and marginally housed populations will help reduce the number of persons needing to use shelters, subsequently decreasing the likelihood of TB exposure at these congregate settings.

Control of TB in homeless populations within Canada will require further progress in primary prevention (e.g., improved ventilation and other infection control measures in shelters), secondary prevention (e.g., earlier detection and treatment of TB infection or disease through greater access to primary care), and tertiary prevention (e.g., treatment of active TB by health care providers with experience treating TB in homeless persons) (*17*). Furthermore, Canada’s interconnectedness with the global community, and consequent interdependence with global TB, necessitates continued vigilance to confront the emerging threat of drug-resistant TB in the world (*40*).
